# Filtration of Public Water Supplies

**Published:** 1906-05

**Authors:** 


FILTRATION OF PUBLIC WATER SUPPLIES.

Cassius E. Gillette names two principal methods which have been used to prepare water for effective filtration with a view to prolonging the "run" and increasing the output. These methods are sedimentation and preliminary filtration or "scrubbing." It is still a matter of experiment for any particular water as to which is the best plan. The determination of this point greatly increases the complexities of engineering design. From various experiments at Lower and at Upper Roxboro, preliminary filtration seems to give rather the better results in summer and sedimentation in the spring. This difference is not marked in the fall and winter. It can be said in general about the Schuylkill water that the removal of the mud improves the water for filtration, partly by lessening the sediment, partly by removing the excess of bacteria, and, perhaps, partly by the improvement of the water in some other respect still undetermined. The writer suggests that it is the part of wisdom for every municipality which takes its supplly of water from streams or lakes to inaugurate small testing plants in order to determine the best way of purifying its water supply.—*Medical Record,* March 24, 1906

